# 8.P. Skills building seminar: Design thinking: adopting a human-centred approach to tackling complex problems

**DOI:** 10.1093/eurpub/ckac129.531

**Published:** 2022-10-25

**Authors:** Organised by: University College Dublin, Chair persons: Kate Frazer (Ireland), Thilo Kroll (Ireland)

## Abstract

The concept of wicked problems is recognised since the 1970s, and social determinants of health (SDOH) account for various difficulties. Fixing or solving wicked problems is challenging due to the complexity. The diverse nature of problems can mean that a solution for one group may result in unintended consequences for others. Healthcare itself is a complex adaptive system. An intersectionality lens may assist in navigating ‘the messiness,’ to reduce inequity and enable voices of those experiencing problems to be involved. COVID-19 amplified difficulties of marginalised populations including ‘othering’. Homelessness is a complex global public health challenge, with limited reliable prevalence data and no universally agreed definition. Being homeless is associated with negative impacts on health and wellbeing, and an absence of belonging. Considering solutions requires a problem-solving focus. Design thinking is a human-centric problem-solving approach focusing on empathy, brainstorming ideas, and prototyping iterative solutions. Equity focused design thinking is a framework to develop people-centred solutions and reflect on the influence of own biases and beliefs on systems. Acknowledging the theme of the conference ‘Strengthening health systems: improving population health and being prepared for the unexpected’ we propose a 60-minute skills-building workshop. We will use a live design sprint format to facilitate collaborative learning for attendees, as an alternative to oral presentations, to explore the intersectionality of design thinking and equity using the Stanford D model. The value of this workshop is knowledge generation and dissemination using an equity lens to adopt creative methods for engagement to produce end-user solutions.

**Objectives:**

– To introduce design thinking approaches to consider solutions for homelessness.

– To facilitate an experiential approach to support learning and collaboration.

– To explore the application of design thinking using an equity focus as a tool to challenge mindsets and create problem-solving in public health.

**Format:**

Working in groups use Stanford D model equity-centred framework (Notice, Empathize, Define, Ideate, Prototype, Test, Reflect).

Introduction (10 min)

– Homelessness ‘a wicked problem'

– Design thinking and equity focus explanation and plan for session

Phases of Design thinking (30-40 min)

– Noticing: awareness of own values, biases, assumptions

– Empathizing: Understanding homelessness from the perspective of the user

– Defining: The specific user challenge

– Ideate: Brainstorm solutions

– Prototype and test: Propose and pitch low fidelity solutions

Final reflection & summary (10 min)

– Insights and learning, networking, and collaboration.

**Key messages:**

• Equity focused design thinking methods provide approaches to increase awareness of the impact of biases and beliefs.

• Human-centred design is an agile approach to creating service and systems developments for marginalised populations.

